# In this month’s *Bulletin*

**DOI:** 10.2471/BLT.15.001215

**Published:** 2015-12-01

In the editorial section, Margaret Chan (818) argues that WHO must deliver a timely response when disease outbreaks and emergencies occur. Rokho Kim et al. (819) claim that a Paris climate pact, if ambitious and effective, should also be regarded as a public health treaty to protect vulnerable populations. 

In the news section, Andréia Azevedo Soares and Menelaos Tzafalias report on how European countries are struggling to respond to the health needs of large numbers of refugees and migrants (822–823). David Nabarro talks to Fiona Fleck about WHO’s transformation into an agency that is fully mandated and equipped to respond to humanitarian emergencies (824–825). 

## Brazil

**Reaching children with congenital heart disease**

Sandra da Silva Mattos et al. (881–887) discuss the use of telemedicine networks for remote paediatric cardiology services. 

## Cambodia

### Preventing unwanted pregnancies

Chris Smith et al. (842–850) examine the effect of a mobile-phone based intervention on contraceptive use. 

## China

### Deaths associated with tuberculosis

Weibing Wang et al. (826–833) estimate excess mortality in a cohort of people with tuberculosis. 

## Sierra Leone

### Alleviating war trauma

Ryan K McBain et al. (834–841) investigate the benefits to caregivers of a psychotherapeutic intervention for young people affected by war. 

## Global 

### Rapid tests for malaria

Theodoor Visser et al. (862–866) discuss trends and developments in diagnostic devices. 

### Influencing smoking habits

Emily Savell et al. (851–861) document exposure to tobacco advertising in 16 countries. 

**Migrants’ health needs**

Lara Miramontes et al. (888–889) call for the inclusion of migrant populations in health impact assessments. 

**Environmental impacts of tobacco **

Thomas E Novotny et al. (877–880) highlight the environmental impacts of tobacco production and use. 

### Antimicrobial resistance 

Steven J Hoffman et al. (867–876) examine ways to achieve global collective action on antimicrobial resistance. 

